# Do symptom-based questions help screen COPD among Chinese populations?

**DOI:** 10.1038/srep30419

**Published:** 2016-07-26

**Authors:** Qun Zhang, Min Wang, Xiaona Li, Hong Wang, Jianming Wang

**Affiliations:** 1 Department of Respiratory, the First Affiliated Hospital of Nanjing Medical University, Nanjing, 210029, China; 2 Health Management Center, the First Affiliated Hospital of Nanjing Medical University, Nanjing, 210029, China; 3 Department of Epidemiology, School of Public Health, Nanjing Medical University, Nanjing, 211166, China; 4 Department of Social Medicine and Health Education, School of Public Health, Nanjing Medical University, Nanjing, 211166, China

## Abstract

Spirometry is required to confirm a chronic obstructive pulmonary disease (COPD) diagnosis, but it is difficult to perform in resource-limited settings. This study aimed to evaluate symptom-based questions for screening of individuals with COPD among Chinese populations. We recruited 3969 adult subjects from the First Affiliated Hospital of Nanjing Medical University. Spirometric measurements of forced expiratory volume in 1 second (FEV1) and forced vital capacity (FVC) were collected to confirm the COPD diagnosis. A symptom-based questionnaire was administered to collect data related to COPD. The sensitivity and specificity together with the area under the curve (AUC) were calculated. The traditional IPAG eight-item questionnaire yielded an AUC of 0.80(95% CI: 0.78–0.82), with a sensitivity of 67.8% and specificity of 76.8%. After removing and adding questions, a revised eleven-item questionnaire exhibited a significantly increased diagnostic accuracy, with an AUC of 0.85(95% CI: 0.84–0.87). At the inflection point of the curve, it demonstrated a sensitivity of 82.5% and specificity of 72.9%. We showed that the revised symptom-based questionnaire could be used to screen individuals with a high likelihood of COPD among Chinese populations. Further validation is required before we claim it is a useful diagnostic for primary care populations.

Chronic obstructive pulmonary disease (COPD) is a common but preventable disorder characterized by chronic inflammation of the airways and a persistently limited airflow that progressively worsens with age^1^,^2^,^3^. It has been predicted that it will be the fifth most common disease and third-leading cause of death worldwide by 2020 4. In China, over 90% of respiratory-related deaths and disability-adjusted life years for adults were attributed to COPD, with most occurring at or over the age of 60 5. COPD is a silent and unrecognized disease in its early phases, causing it to be generally underdiagnosed^6^,^7^,^8^,^9^. At least 328 million people are estimated to suffer from COPD throughout the world; however, 80% or more cases are not detected on time and, hence, are not treated^10^. This underdiagnoses results from patients being unaware of the relevant risk factors and failing to recognize related symptoms; thus, they do not seek health care until the disease has progressed^11^,^12^. Among physicians, failure to suspect and test for COPD leads to inappropriate treatments^13^,^14^.

Spirometry is required to confirm a COPD diagnosis^1^,^15^; but it is difficult to perform in resource-limited settings and is often regarded as difficult for the patient, complex to interpret, time-consuming and cumbersome^16^,^17^. The handbook drafted by the International Primary Care Airway Group (IPAG) recommends the use of a symptoms-based questionnaire with guidelines to assist primary care physicians in diagnosing and treating chronic airway diseases, such as asthma, COPD and allergic rhinitis^18^. Such symptoms-based questionnaires are easy, feasible, economic and effective relative to spirometry examinations. Previous studies have shown that using spirometry in combination with a symptom-based questionnaire can significantly improve the diagnostic accuracy of obstructive lung diseases^16^.

However, the commonly used eight-item questionnaire, which was originally developed by Price *et al*.^13^, was mainly validated in Western countries^18^,^19^, and, populations from the developing countries may be inadequately assessed by this questionnaire. We hypothesize that the original eight-item questionnaire needs to be modified for the Chinese population. Thus, we performed an epidemiological study to evaluate the effectiveness and feasibility of a revised symptom-based questionnaire to screen Chinese patients with COPD.

## Materials and Methods

### Study subjects

A cross-sectional study was performed at the First Affiliated Hospital of Nanjing Medical University, China in 2014. We recruited 3969 adult subjects aged over 30 years from the Division of Respiratory Medicine or the Health Management Center. We designed a symptom-based questionnaire by referring to the traditional eight-item questionnaire^13^,^19^, literature on COPD among the Chinese population and expert opinions from the First Affiliated Hospital of Nanjing Medical University. The questions included age, tobacco smoking, coughing, sputum, wheezing, dyspnea and other early symptoms of COPD or potential risk factors. Trained staff explained the questionnaire and provided assistance to all study subjects. Written informed consent was obtained from each subject before enrollment. This project was approved by the Institutional Review Board of Nanjing Medical University. The methods were carried out in accordance with the approved guidelines.

### Physical measurements

The body weight of participants wearing a light dressing gown was measured. The body mass index (BMI) was calculated from the weight in kilograms divided by the height in meters squared. After an eligibility evaluation, the indicated subjects underwent spirometry. The dynamic lung volumes were performed using a standard spirometer to measure the forced expiratory volume in 1 second (FEV1) and forced vital capacity (FVC)^20^. We determined a quality grade (A–F) based on the acceptable maneuvers and repeatability of the FEV1 and FVC. Those with spirometry results of grades A, B, or C were considered acceptable for analysis. If subjects had suspicious airflow limitations (FEV1/FVC < 0.70 or FEV1 < 80% predicted), then post-bronchodilator testing was performed 15 to 20 minutes after inhaling a dose of 200 mg of salbutamol (Ventolin, GlaxoSmithKline, Middlesex, UK) through a 500-ml spacer immediately. All diagnosis was made by the respiratory specialist according to the diagnostic criteria of the Global Initiative for Chronic Obstructive Lung Disease (GOLD). COPD was defined as an FEV1/FVC ratio below 70%. The severity of airway obstruction was graded as follows: stage I, mild, FEV1 ≥ 80% predicted; stage II, moderate, 50% predicted ≤ FEV1 < 80% predicted; stage III, severe, 30% predicted ≤ FEV1 < 50% predicted; and stage IV, extremely severe, FEV1 < 30% predicted^4^.

The spirometer operators included one senior technician and four nurses. All of them had been trained for at least one month in the Lung Function Laboratory at the First Affiliated Hospital of Nanjing Medical University and accredited before the survey. We used two types of spirometers, the Multi-Functional Spirometer HI-801 (CHEST M.I., INC., Japan) that was adopted in the Health Management Center and the Carefusion (MasterScreen, Germany) that was adopted in the Division of Respiratory Medicine. The results of these two types of spirometer were compared and the consistency was over 95%. In the Health Management Center, the spirometer was calibrated monthly with a volume variation of less than 3% by a 3-L syringe. In the Division of Respiratory Medicine, the spirometer was calibrated daily with a volume variation of less than 3% by a 3-L syringe and a three velocity calibration was carried out to inspect the velocity sensor linearity after standard calibration.

### Statistical analysis

The statistical analyses were performed using STATA 10.0 (StataCorp, College Station, TX, USA). Continuous variables were described using either the mean (standard deviation) or median with inter-quartile range (IQR). The categorical variables were described using their frequency and proportion. Subjects were divided into the COPD group and healthy control group based on the spirometry. The Wilcoxon rank-sum test or Kruskal-Wallis rank test were used to compare the scores for two or multiple groups. We used an unconditional logistic regression model to evaluate the relationship between selected factors and the risk of COPD. The strength of the relationship was estimated using the odds ratio (OR) together with the 95% confidence interval (CI). The diagnostic sensitivity, specificity, Youden’s index and likelihood ratio (LR) were calculated. The receiver operating characteristic (ROC) curve was graphed to estimate the diagnostic value of the selected factors. The areas under the curves (AUCs) were calculated and compared via the χ^2^ test. The test level was set at 0.05.

## Results

### General characteristics

We recruited 3969 study subjects, including 2483 men and 1486 women. Of them, 490 were diagnosed with COPD and 3479 were defined as healthy controls. For the COPD cases, 80 (16.3%) were mild, 262 (53.5%) were moderate, 115 (23.5%) were severe, and 33 (6.7%) were extremely severe. The basic demographic characteristics for the study subjects are shown in Table 1. A significant difference existed for the gender, place of residence, education background and per-capita income distribution.

### Accuracy of the eight-item symptom-based questionnaire

**Figure 1:**
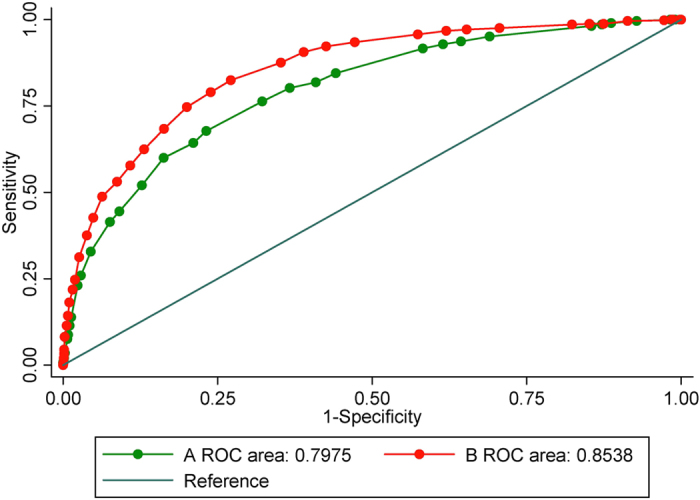
Receiver operating characteristic curve for the symptom-based questionnaire. (A) the traditional eight-item questionnaire; (B) the revised eleven-item questionnaire.

As recommended by IPAGE, different scores were assigned to each questionnaire item. We summed the scores for each subject based on their responses. The scores for COPD cases ranged from 5 to 36 with a median (IQR) of 20 (16–24), while the scores for the healthy controls ranged from 2 to 35 with a median (IQR) of 12 (8–16). The Wilcoxon rank-sum (Mann-Whitney U) test indicated that scores for the COPD group were significantly higher than those for the healthy controls (z = −21.43, P < 0.001). The ROC of the eight-item questionnaire in screening COPD is illustrated in Fig. 1. The AUC was 0.80 (95% CI: 0.78–0.82). If we set the cut-point at 17 as recommended, then the sensitivity and specificity were 67.76% and 76.83%, respectively. The diagnostic values using different cut-points were listed in the Supplementary Table 1.

Table 2 shows the frequency distribution and discriminatory capability of individual items. Of the eight questionnaire items, two (phlegm without a cold, P = 0.795; phlegm in the morning, P = 0.362) had no statistically significant relationship to COPD in the study population. Moreover, we observed that BMI had a non-significant negative correlation with the risk of COPD. If we excluded the items of “phlegm without a cold”, “phlegm in the morning” and “BMI” from the questionnaire, then the AUC increased to 0.82 (95% CI: 0.80–0.84).

### Revision of the symptom-based questionnaire

Considering the different BMI distribution in different countries, we modified the category by referring to the criteria of overweight/obese in the Chinese population, where subjects were classified as obese (BMI >= 28), overweight (BMI: 24–28) and normal (BMI < 24). Moreover, we identified several specific factors relating to the risk of COPD in this study (Table 3). According to their strength of association (OR), we assigned different scores to each response (OR: 1-2, score: 1; OR: 2-3, score: 2; OR: 3-4, score: 3; OR: 4-5, score: 4). As shown in Table 4, 11 items were adopted in the final model. The ROC was illustrated in Fig. 1 with an AUC of 0.85 (95% CI: 0.84–0.87), which was significantly higher than that for the original eight-item questionnaire (χ^2^ = 86.18, P < 0.001). At the inflection point of the curve (score: 17), this questionnaire demonstrated a sensitivity of 82.45% and specificity of 72.87% with a Youden’s index of 55.32% (Table 5). The kappa value was 0.34 (P < 0.001). With an increased cut-point, the specificity increased but the sensitivity declined (Supplementary Table 2).

**Figure 2:**
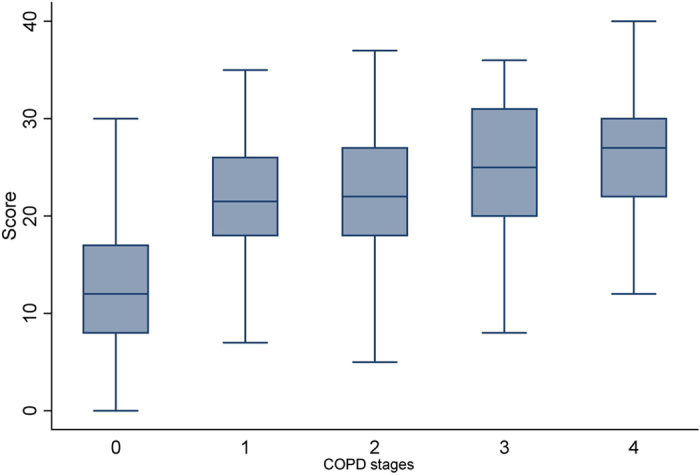
Scores for the revised eleven-item questionnaire for the healthy control group and patients at different COPD stages. The line through the box is the median. The top and bottom edges of each box represent the 25th and 75th percentiles, giving the interquartile range. The vertical lines at each side of the box represent distribution from the quartile to the farthest observation. COPD stages: 0, healthy control; 1, stage I; 2, stage II; 3, stage III; 4, stage IV.

A significant difference existed between the stages of COPD (Kruskal-Wallis rank test, P < 0.001). Higher stages of COPD exhibited increased scores with a correlation coefficient of r = 0.45 (P < 0.001) (Fig. 2).

## Discussion

COPD remains significantly underdiagnosed worldwide, with correct diagnosis commonly missed or delayed until the pulmonary impairment has advanced^21^. In this study, we evaluated an eight-item questionnaire and a revised questionnaire to screen for COPD among Chinese populations. Our results showed that the symptom-based questionnaire was a simple tool to identify individuals with a high likelihood of COPD; however, the items and scores need to be modified for different settings.

COPD is a lung ailment characterized by a persistent airway obstruction of airflow from the lungs and, has become a major global health problem with increasing morbidity and mortality^22^,^23^. Associated with global social and economic burdens, it is estimated to increase in both developed and developing countries due to the continuous use of tobacco and biomass fuels among aging populations^24^. This lung disease is under-diagnosed, life-threatening, obtrusive to normal breathing and not fully reversible^25^. In the later stages, COPD greatly impairs quality of life and leads to repeated costly hospital stays. If it can be prevented, detected and treated early, then the disease progression will be delayed^26^. Underuse of spirometry in the primary care and resource-limited settings contributes to the underdiagnoses of COPD^13^. General practitioners need simple tools to address the question, “Which of my patients are likely to have COPD?”. Implementing a simple, self-administered questionnaire may help identify those for whom spirometric testing is particularly important and encourage timely and appropriate COPD evaluation.

We explored the factors listed in the eight-item questionnaire^13^ using a logistic regression model. As expected, age, smoking pack-years, weather-affected coughing, wheeze frequency and history of allergies were significantly related to COPD. However, BMI, phlegm without a cold and morning phlegm had low discriminatory capabilities. An alternative explanation for the lack of differentiation for questions regarding expectoration is that airway reactivity and secretions increase among most Chinese people due to long-term smoking, environmental tobacco smoke (ETS), recurrent respiratory tract infections during childhood and deteriorating air quality^27^,^28^,^29^,^30^. A relatively high prevalence of phlegm in the Chinese population may limit its discriminatory capability for COPD^31^. BMI, a prognostic factor for COPD, was used to evaluate a person’s pulmonary function, indicated an association between decreasing body mass and increasing mortality and may have been set too high for Chinese subjects^32^. Thus, we modified the categories of BMI according to Chinese criteria and yielded significant associations as expected.

Using 17 as a cut-point value for the original eight-item questionnaire yielded a sensitivity of 67.8%, which was lower than that reported in Western countries (80.4%) and higher than that in Japan (14.3%); however, the specificity (76.8%) was higher than that reported by Price *et al*. (57.5%) but lower than that reported by Arimura *et al*. (83.2%)^19^,^33^. This may be attributable to the physical, ethnic and geographical disparities.

In this study, we selected several specific factors and explored their associations with COPD among the Chinese population. For example, the COPD group was exposed to more dust or chemical particles than the healthy controls, which suggested this factor may be an alternative factor for COPD screening^34^,^35^. Studies have proven tobacco smoking is the main risk factor for COPD^36^,^37^. We found that exposure to second-hand smoke was also related to COPD. Adding this item to the questionnaire can help increase the diagnostic accuracy. By adding and removing questions, we constructed an 11-item questionnaire to screen for COPD among the Chinese population. This yielded an AUC of 0.85 (95% CI: 0.84–0.87), which was significantly higher than that for the original eight-item questionnaire. At the cut-point of 17, this questionnaire demonstrated a sensitivity of 82.45% and specificity of 72.87%.

This study has certain limitations. First, it was performed at the First People’s Hospital of Nanjing Medical University in Jiangsu Province. Our subjects volunteered to participate and may not reflect the general nature of primary care practices. The proportion of respondents with COPD should not be used to estimate the population prevalence and it remains unclear how applicable the described findings are to the primary care population. These results need to be replicated in unselected health care settings. Second, because this was a cross-sectional study, our results could have been impacted by practices that affect which patients received a respiratory diagnosis or respiratory illness medication. Third, this was only a preliminary report using a revised symptom-based questionnaire to identify COPD in China. Creating more sensitive and specific questions and assigning appropriate response points based on the situation in China is necessary going forward.

## Conclusions

A revised symptom-based questionnaire can be used to identify Chinese patients with a high likelihood of having COPD. Developing a more sensitive and specific questionnaire will require additional studies, including a prospective validation of items in an appropriate clinical setting and policy recommendations for using these tools.

## Additional Information

**How to cite this article**: Zhang, Q. *et al*. Do symptom-based questions help screen COPD among Chinese populations? *Sci. Rep.* **6**, 30419; doi: 10.1038/srep30419 (2016).

## Supplementary Material

Supplementary Information

## Figures and Tables

**Table 1:** Basic demographic characteristics of study subjects.

Variable	Total (n = 3969)	Control (n = 3479)	COPD (n = 490)	χ^2^	P
Gender					
Men	2483(62.6)	2135(61.4)	348(71.0)	17.08	<0.001
Women	1486(37.4)	1344(38.6)	142(29.0)		
Resident area					
Suburb	587(14.8)	486(14.0)	101(20.6)	126.12	<0.001
City	2764(69.6)	2524(72.6)	240(49.0)		
Suburban junction	618(15.6)	469(13.5)	149(30.4)		
Education					
Illiterate	473(11.9)	345(9.9)	128(26.1)	231.77	<0.001
Primary school	351(8.8)	288(8.3)	63(12.9)		
Junior high school	552(13.9)	458(13.2)	94(19.2)		
Senior high school	837(21.1)	706(20.3)	131(26.73)		
College or over	1756(44.2)	1682(48.4)	74(15.1)		
Income per month*, RMB (USD)					
<2001(309)	672(16.9)	523(15.0)	149(30.4)	86.59	<0.001
2001(309) -	289(7.3)	246(7.1)	43(8.8)		
3001(464) -	298(7.5)	253(7.3)	45(9.2)		
4001(618) -	457(11.5)	412(11.8)	45(9.2)		
5001(772) -	2253(56.8)	2045(58.8)	208(42.5)		
COPD stage					
I, mild	—	—	80(16.3)		
II, moderate	—		262(53.5)		
III, severe	—	—	115(23.5)		
IV, extremely severe	—	—	33(6.7)		

**Table 2:** Comparison of responses to the eight-item questionnaire by COPD patients and controls.

Variable	Control (n = 3479)	COPD (n = 490)	χ^2^	*P*	*OR (95% CI)*	*P*
Age (years)						
<50	1586(45.6)	71(14.5)	410.3	<0.001	1	
50-	940(27.0)	86(17.6)			2.04(1.48–2.83)	<0.001
60-	667(19.2)	166(33.9)			5.56(4.15–7.45)	<0.001
70-	286(8.2)	167(34.1)			13.04(9.62–17.69)	<0.001
Smoking (pack-years)						
<14	2694(77.4)	237(48.4)	254.13	<0.001	1	
15-	326(9.4)	54(11.0)			1.88(1.37–2.59)	<0.001
25-	367(10.6)	144(29.4)			4.46(3.53–5.64)	<0.001
50-	92(2.6)	55(11.2)			6.80(4.74–9.74)	<0.001
BMI (kg/m^2^)						
<25.4	2308(66.3)	355(72.5)	7.28	0.026	1.37(0.88–2.15)	0.158
25.4–	965(27.7)	112(22.9)			1.04(0.65–1.67)	0.872
29.7-	206(5.9)	23(4.7)			1	
Does the weather affect cough						
Yes	394(11.3)	85(17.4)	14.68	<0.001	1.64(1.27–2.12)	<0.001
No	3085(88.7)	405(82.6)			1	
Phlegm without a cold						
Yes	545(15.7)	79(16.1)	0.07	0.795	1.03(0.80–1.34)	0.795
No	2934(84.3)	411(83.9)			1	
Phlegm in the morning						
Yes	471(13.5)	59(12.0)	0.83	0.362	1	
No	3008(86.5)	431(88.0)			0.87(0.65–1.17)	0.362
Wheeze frequency						
Never	2266(65.1)	81(16.5)	419.85	<0.001	1	
Occasionally or more often	1213(34.9)	409(83.5)			9.43(7.36–12.09)	<0.001
Allergic history						
Yes	567(16.3)	59(12.0)	5.86	0.015	1	
No	2912(83.7)	431(88.0)			1.42(1.07–1.89)	0.016

**Table 3:** Other potential factors related to the risk of COPD.

Variables	Control (n = 3479)	COPD (n = 490)	*OR (95% CI)*	*P*
Exposure to the second-hand smoking (hrs/week)				
<7	2955(84.9)	387(79.0)	1.50(1.18–1.90)	0.001
7-	524(15.1)	103(21.0)		
Cough without a cold				
No	3091(88.9)	419(85.5)	1	
Yes	388(11.2)	71(14.5)	1.35(1.03–1.77)	0.031
Shortness of breath				
No	2530(72.7)	178(36.3)	1	
Yes	949(27.3)	312(63.7)	4.67(3.83–5.70)	<0.001
Long-term exposure to dust or chemical particles				
No	2964(85.2)	303(61.8)	1	
Yes	515(14.8)	187(38.2)	3.55(2.89–4.36)	<0.001
History of chronic respiratory diseases during childhood				
No	3174(91.2)	349(71.2)	1	
Yes	305(8.8)	141(28.8)	4.20(3.35–5.28)	<0.001
BMI (kg/m^2^)				
28-	419(12.04)	48(9.8)	1	
24-	1357(39.01)	164(33.47)	1.05(0.75–1.48)	0.758
<24	1703(48.95)	278(56.73)	1.42(1.03–1.97)	0.032

**Table 4:** Symptom-based questionnaire for screening COPD.

Question	Original questionnaire		Revised questionnaire	
	Response choices	Points	Response choices	Points
1. What is your age in years?	40–49 years	0	40–49 years	0
	50–59 years	4	50–59 years	4
	60–69 years	8	60–69 years	8
	70 years or older	10	70 years or older	10
2. How many cigarettes do you currently smoke each day (if you are an ex-smoker, how many did you smoke each day)? What is the total number of years you have smoked cigarettes? Packs per day = cigarettes per day/20 per pack Pack-years = packs per day × years smoked	0–14 pack-years	0	0–14 pack-years	0
	15–24 pack-years	2	15–24 pack-years	2
	25–49 pack-years	3	25–49 pack-years	3
	50 + pack-years	7	50 + pack-years	7
3. What is your weight in kilograms? What is your height in meters? BMI = weight in kg/(height in m)^2^	BMI < 25.4	5	BMI < 24	5
	BMI 25.4–29.7	1	BMI 24–28	1
	BMI > 29.7	0	BMI >= 28	0
4. Does weather affect your cough?	Yes	3	Yes	3
	No	0	No	0
5. Do you ever cough up phlegm (sputum) from your chest when you do not have a cold?	Yes	3		
	No	0		
6. Do you usually cough up phlegm (sputum) from your chest first thing in the morning?	Yes	0		
	No	3		
7. How frequently do you wheeze?	Never	0	Never	0
	Occasionally or more often	4	Occasionally or more often	4
8. Do you have or have you had any allergies?	Yes	0	Yes	0
	No	3	No	3
9. How frequently are you exposed to second-hand smoking?			<7 hrs/week	0
			>=7 hrs/week	1
10. Do you often cough when you do not have a cold?			Yes	1
			No	0
11. Do you have more signs of shortness of breath compared with others of the same age?			Yes	4
			No	0
12. Have you had long-term exposure to dust or chemical particles?			Yes	3
			No	0
13. Did you have a history of chronic respiratory diseases when you were a child?			Yes	4
			No	0

**Table 5:** Diagnostic accuracy of the revised symptom-based questionnaire.

Diagnostic test*	Value (%)	95% CI (%)
Sensitivity	82.45	78.79–85.71
Specificity	72.87	71.36–74.34
Positive predictive value (PPV)	29.97	27.53–32.49
Negative predictive value (NPV)	96.72	95.96–97.37
Kappa	0.32	
