# Supplementary material for: Do symptom-based questions help screen COPD among Chinese populations?
Source: Sci Rep. 2016 Jul 26;6:30419. doi: 10.1038/srep30419 (PMC4960647; doi:10.1038/srep30419)
Supplement: Supplementary Information [file srep30419-s1.pdf]

# Do symptom-based questions help screen COPD among Chinese populations?

Qun Zhang[1,2*], Min Wang[3*], Xiaona Li[2], Hong Wang[1 §], Jianming Wang[3,4 §]

1Department of Respiratory, the First Affiliated Hospital of Nanjing Medical University, Nanjing, 210029, China

2Health Management Center, the First Affiliated Hospital of Nanjing Medical University, Nanjing, 210029, China

3Department of Epidemiology, School of Public Health, Nanjing Medical University, Nanjing, 211166, China

4Department of Social Medicine and Health Education, School of Public Health, Nanjing Medical University, Nanjing, 211166, China

[*]These authors contributed equally to this work

[§]Corresponding author

Supplement Table 1. The diagnostic accuracy of the previous eight-item questionnaire

| Cut-point | Sensitivity (%) | Specificity (%) | Youden's index | LR+ | LR- |
|---|---|---|---|---|---|
| >= 0 | 100.00 | 0.00 | 0.00 | 1 | |
| >= 1 | 100.00 | 0.17 | 0.17 | 1.0017 | 0 |
| >= 2 | 100.00 | 0.95 | 0.95 | 1.0096 | 0 |
| >= 3 | 100.00 | 1.06 | 1.06 | 1.0107 | 0 |
| >= 4 | 100.00 | 1.75 | 1.75 | 1.0178 | 0 |
| >= 5 | 99.59 | 7.19 | 6.78 | 1.073 | 0.0568 |
| >= 6 | 98.98 | 11.33 | 10.31 | 1.1162 | 0.0901 |
| >= 7 | 98.57 | 12.82 | 11.39 | 1.1307 | 0.1114 |
| >= 8 | 98.16 | 14.52 | 12.68 | 1.1483 | 0.1265 |
| >= 9 | 95.10 | 30.99 | 26.09 | 1.378 | 0.1581 |
| >= 10 | 93.67 | 35.61 | 29.28 | 1.4549 | 0.1776 |
| >= 11 | 92.86 | 38.52 | 31.38 | 1.5103 | 0.1854 |
| >= 12 | 91.63 | 41.79 | 33.42 | 1.5743 | 0.2002 |
| >= 13 | 84.49 | 55.88 | 40.37 | 1.9149 | 0.2776 |
| >= 14 | 81.84 | 59.10 | 40.94 | 2.0008 | 0.3073 |
| >= 15 | 80.20 | 63.32 | 43.52 | 2.1868 | 0.3126 |
| >= 16 | 76.33 | 67.78 | 44.11 | 2.3688 | 0.3493 |
| >= 17 | 67.76 | 76.83 | 44.59 | 2.9246 | 0.4197 |
| >= 18 | 64.29 | 78.93 | 43.22 | 3.0512 | 0.4525 |
| >= 19 | 60.00 | 83.73 | 43.73 | 3.688 | 0.4777 |
| >= 20 | 52.04 | 87.24 | 39.28 | 4.0777 | 0.5498 |
| >= 21 | 44.49 | 90.89 | 35.38 | 4.8826 | 0.6108 |
| >= 22 | 41.43 | 92.41 | 33.84 | 5.4595 | 0.6338 |
| >= 23 | 32.86 | 95.52 | 28.38 | 7.3276 | 0.7029 |
| >= 24 | 25.92 | 97.13 | 23.05 | 9.017 | 0.7627 |
| >= 25 | 23.06 | 97.64 | 20.70 | 9.7841 | 0.788 |
| >= 26 | 13.88 | 98.68 | 12.56 | 10.4956 | 0.8728 |
| >= 27 | 11.43 | 98.94 | 10.37 | 10.746 | 0.8952 |
| >= 28 | 8.78 | 99.20 | 7.98 | 10.9035 | 0.9196 |
| >= 29 | 7.55 | 99.31 | 6.86 | 10.9459 | 0.9309 |
| >= 30 | 3.47 | 99.71 | 3.18 | 12.0701 | 0.9681 |
| >= 31 | 2.04 | 99.83 | 1.87 | 11.8334 | 0.9813 |
| >= 32 | 1.84 | 99.89 | 1.73 | 15.9751 | 0.9828 |
| >= 33 | 1.22 | 99.91 | 1.13 | 14.2007 | 0.9886 |
| >= 34 | 0.82 | 99.97 | 0.79 | 28.4039 | 0.9921 |
| >= 35 | 0.61 | 99.97 | 0.58 | 21.3029 | 0.9942 |
| >= 36 | 0.61 | 100.00 | 0.61 | | 0.9939 |
| > 36 | 0.00 | 100.00 | 0.00 | | 1 |

Supplement Table 2. The diagnostic accuracy of the revised symptom-based questionnaire

| Cut-point | Sensitivity (%) | Specificity (%) | Youden's index | LR+ | LR- |
|---|---|---|---|---|---|
| >= 0 | 100.00 | 0.00 | 0.00 | 1 | |
| >= 1 | 100.00 | 0.17 | 0.17 | 1.0017 | 0 |
| >= 2 | 100.00 | 1.32 | 1.32 | 1.0134 | 0 |
| >= 3 | 100.00 | 1.49 | 1.49 | 1.0152 | 0 |
| >= 4 | 99.80 | 2.76 | 2.56 | 1.0263 | 0.074 |
| >= 5 | 99.59 | 8.65 | 8.24 | 1.0902 | 0.0472 |
| >= 6 | 98.78 | 12.53 | 11.31 | 1.1293 | 0.0977 |
| >= 7 | 98.78 | 14.83 | 13.61 | 1.1598 | 0.0826 |
| >= 8 | 98.57 | 17.65 | 16.22 | 1.197 | 0.0809 |
| >= 9 | 97.55 | 29.38 | 26.93 | 1.3813 | 0.0834 |
| >= 10 | 97.14 | 34.69 | 31.83 | 1.4875 | 0.0824 |
| >= 11 | 96.73 | 38.00 | 34.73 | 1.5602 | 0.0859 |
| >= 12 | 95.71 | 42.60 | 38.31 | 1.6675 | 0.1006 |
| >= 13 | 93.47 | 52.80 | 46.27 | 1.9804 | 0.1237 |
| >= 14 | 92.24 | 57.43 | 49.67 | 2.1669 | 0.135 |
| >= 15 | 90.61 | 61.05 | 51.66 | 2.3265 | 0.1538 |
| >= 16 | 87.55 | 64.76 | 52.31 | 2.4844 | 0.1922 |
| >= 17 | 82.45 | 72.87 | 55.32 | 3.0386 | 0.2409 |
| >= 18 | 78.98 | 76.11 | 55.09 | 3.3065 | 0.2762 |
| >= 19 | 74.69 | 79.97 | 54.66 | 3.7283 | 0.3165 |
| >= 20 | 68.37 | 83.67 | 52.04 | 4.1875 | 0.378 |
| >= 21 | 62.45 | 86.89 | 49.34 | 4.7645 | 0.4322 |
| >= 22 | 57.76 | 89.13 | 46.89 | 5.3156 | 0.4739 |
| >= 23 | 53.06 | 91.26 | 44.32 | 6.0724 | 0.5143 |
| >= 24 | 48.78 | 93.68 | 42.46 | 7.7132 | 0.5468 |
| >= 25 | 42.65 | 95.11 | 37.76 | 8.7288 | 0.6029 |
| >= 26 | 37.55 | 96.15 | 33.70 | 9.7492 | 0.6495 |
| >= 27 | 31.22 | 97.38 | 28.60 | 11.9373 | 0.7062 |
| >= 28 | 24.69 | 98.07 | 22.76 | 12.8224 | 0.7678 |
| >= 29 | 21.84 | 98.45 | 20.29 | 14.0685 | 0.794 |
| >= 30 | 18.16 | 98.99 | 17.15 | 18.0543 | 0.8267 |
| >= 31 | 14.29 | 99.22 | 13.51 | 18.4074 | 0.8638 |
| >= 32 | 11.43 | 99.43 | 10.86 | 19.8801 | 0.8908 |
| >= 33 | 8.16 | 99.74 | 7.90 | 31.5553 | 0.9207 |
| >= 34 | 4.49 | 99.83 | 4.32 | 26.0335 | 0.9568 |
| >= 35 | 3.27 | 99.83 | 3.10 | 18.9334 | 0.969 |
| >= 36 | 2.04 | 99.89 | 1.93 | 17.7501 | 0.9807 |
| >= 37 | 0.61 | 99.94 | 0.55 | 10.6501 | 0.9944 |
| >= 40 | 0.20 | 100.00 | 0.20 | | 0.998 |
| > 40 | 0.00 | 100.00 | 0.00 | | 1 |
